# Weighted single-step genomic best linear unbiased predictor enhances the genomic prediction accuracy for milk citrate predicted by milk mid-infrared spectra of Holstein cows in early lactation

**DOI:** 10.3168/jdsc.2024-0607

**Published:** 2024-09-27

**Authors:** Y. Chen, H. Atashi, C. Grelet, N. Gengler

**Affiliations:** 1 TERRA Teaching and Research Center, University of Liège, Gembloux Agro-Bio Tech (ULiège-GxABT), 5030 Gembloux, Belgium; 2 Department of Animal Science, Shiraz University, 71441-65186 Shiraz, Iran; 3 Walloon Agricultural Research Center (CRA-W), 5030 Gembloux, Belgium

## Abstract

Previous studies have shown that milk citrate predicted by milk mid-infrared (MIR) spectra is strongly affected by a few genomic regions. This study aimed to explore the effect of weighted single-step GBLUP on the accuracy of genomic prediction (GP) for MIR-predicted milk citrate in early-lactation Holstein cows. A total of 134,517 test-day predicted milk citrate collected within the first 50 DIM on 52,198 Holstein cows from the first 5 parities were used. There were 122,218 animals in the pedigree, of which 4,479 had genotypic data for 566,170 SNPs. Two datasets (partial and whole datasets) were used to verify whether the accuracy of GP is improved using the following different methods. The (genomic) estimated breeding values (EBV or GEBV) in the partial and whole datasets were estimated by pedigree-based BLUP (ABLUP), single-step GBLUP (ssGBLUP, pedigree-genomic combined using no weight for SNP), and weighted ssGBLUP (WssGBLUP, pedigree-genomic combined using weighted SNP), respectively. The difference between the 2 datasets is that the phenotypic data from 2017 to 2019 in the partial dataset were set as missing values. One hundred eighty-one youngest cows with genomic data were selected as the validation population. A linear regression method was used to compare EBV (GEBV) predicted for partial and whole datasets. The accuracies of GP for ABLUP and ssGBLUP were 0.42 and 0.70, respectively. The accuracies of GP for WssGBLUP in the 5 iterations with different CT (constant) values (determines departure from normality for SNP effects) ranged from 0.70 to 0.86. This study showed that weighted SNP is beneficial in improving prediction accuracy for predicted milk citrate.

## Highlights

• Genomic information facilitates the genomic selection of milk citrate.
• Weighted genomic information improves the genomic prediction accuracy of milk citrate.
• The results of weighted ssGBLUP are affected by the number of iterations.

## Graphical Abstract

**Figure:**
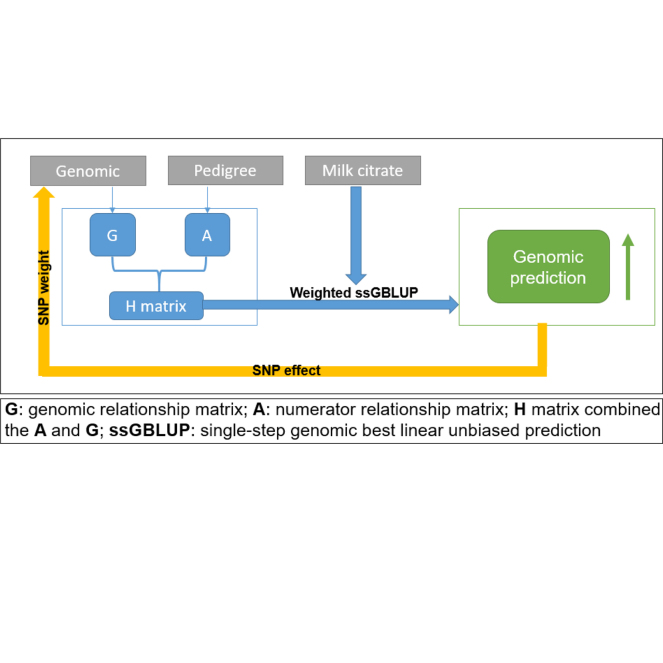
**Summary:** Previous studies have shown that milk citrate predicted by milk mid-infrared spectra is strongly affected by a few genomic regions. The prediction accuracies of pedigree-based best linear unbiased predictor (ABLUP), single-step genomic best linear unbiased predictor (ssGBLUP), and weighted ssGBLUP (WssGBLUP) were compared by linear regression. The prediction accuracies of WssGBLUP (ranging from 0.70-0.86 for different iterations) were higher than those of ABLUP (0.42) and ssGBLUP (0.70). This study showed that weighted SNP is beneficial in improving genomic prediction accuracy for predicted milk citrate.

Genomic prediction (**GP**) is widely used in animal breeding. The methods used for GP in genomic evaluation are currently divided into 2 main classes: Bayesian (Meuwissen et al., 2001) and BLUP (including SNP-BLUP, genomic BLUP [**GBLUP**], and single-step GBLUP [**ssGBLUP**]) methods. Best linear unbiased predictor can perform the calculations more efficiently and is widely used in genomic evaluation in various countries, especially ssGBLUP (Legarra et al., 2009; Bermann et al., 2022). However, the advantage of Bayesian algorithms is that these methods consider the different variance of each SNP effect. The ssGBLUP assumes that all SNP effects have the same variance, which is inconsistent with some real-life examples of traits affected by major genes (e.g., double-muscled in cattle, Grobet et al., 1997). Therefore, Wang et al. (2012) proposed the weighted ssGBLUP (**WssGBLUP**) approach, which assigns different weights to SNPs to construct a new relationship matrix. Lourenco et al. (2017) showed that WssGBLUP is more effective when the number of genotyped individuals is small, and few QTL affect traits. Local breeds or novel traits (e.g., methane production) often have genomic data for only a few animals. Therefore, WssGBLUP may enhance the accuracy of GP for the local breeds or novel traits.

Negative energy balance (**NEB**) is a condition encountered by almost all high-producing dairy cows during early lactation. Negative energy balance is detrimental to the reproduction, metabolism, and infectious diseases of dairy cows (Walsh et al., 2011; Zachut et al., 2020), which may cause economic losses to farmers and reduce the welfare of dairy cows. However, direct measurement of NEB is challenging, prompting researchers to use blood or milk biomarkers to assess the energy status of dairy cows (Zachut et al., 2020). Blood nonesterified fatty acids have been demonstrated as biomarkers for detecting NEB (Zachut et al., 2020), but testing them is very expensive. Milk citrate is proposed as a novel biomarker of NEB and can be applied on a large scale through milk mid-infrared (**MIR**) spectra (Grelet et al., 2016, 2024). Our recent study showed that a few genomic regions have large effects on MIR-predicted citrate (Chen et al., 2024), consistent with other studies in different breeds (Sanchez et al., 2021).

This study aimed to investigate whether WssGBLUP improves the accuracy of GP for MIR-predicted citrate of Holstein cows in early lactation.

The data and model used for this study came from our recent work (Chen et al., 2024). Briefly, 134,517 MIR-predicted citrates (hereafter called citrate) from the first 5 parities of 52,198 Holstein cows in 774 farms in the Walloon Region of Belgium were used. The citrate prediction model was based on the standardized milk MIR spectra extracted from the official milk record database of the Walloon Region of Belgium. The coefficient of determination and root mean square error of validation for the citrate equation were 0.86 and 0.07 mmol/L, respectively (Grelet et al., 2016). The used citrate data were limited to the early lactation (first 50 DIM), a period in which most high-yielding Holstein cows are in NEB (Churakov et al., 2021). The used pedigree includes 122,218 animals, of which 4,479 (3,215 cows and 1,264 bulls) had data for 566,170 SNPs. Citrate in the first 5 parities was considered as one trait based on our latest research (Chen et al., 2024). Hence, a univariate repeatability model was employed to estimate both the variance components and (genomic) estimated breeding values (EBV or **GEBV**) for citrate. The model incorporated fixed effects: herd-year-season of calving, standardized DIM and its quadratic term, and standardized calving age with constant, linear, and quadratic regression (nested within parities); random effects included permanent environmental effects, additive animal genetic effects, and residual effects. To calculate the relationship matrix, either a single (**H**) or pedigree-based **(A**) relationship matrix was employed. The **H** matrix combined the **A** and genomic (**G**)-based relationship matrices, and then **H** was inversed by the method proposed by Aguilar et al. (2010). **A** is the numerator relationship matrix for all animals included in the pedigree; **G** is the genomic relationship matrix of genotyped animals obtained using the first formula described by VanRaden (2008):

$$G=\frac{ZDZ{\;}^{\prime }}{2\sum_{i=1}^{N}\;p_{i}\left(1-p_{i}\right)}=\;ZDZ{\;}^{\prime }\lambda ,$$

where **Z** is a matrix of gene content adjusted for allele frequencies (0, 1, or 2 for aa, Aa, and AA, respectively); **D** is a diagonal matrix of SNP weight; N is the number of SNPs; *p_i_* is the minor allele frequency of the *i*th SNP; *λ* is $\frac{1}{2\sum_{i=1}^{N}\;p_{i}\left(1-p_{i}\right)}.$ If **D** matrix is equal to the identity matrix, the combined relationship matrix (**A** and **G**) is the normal **H** matrix (ssGBLUP); if the diagonal of the **D** matrix is not equal to 1 (SNP weight), the combined relationship matrix (**A** and **G**) is the weighted **H** matrix (WssGBLUP). The SNP weight was calculated based on the procedure proposed by Wang et al. (2012); however, the nonlinear A weights method (VanRaden, 2008) was used in this study. The algorithm proceeds as follows:

(1) *t* = 1, **D**_(_*_t_*_)_ = **I**, $G_{\left(t\right)}^{*}$ = $ZD_{\left(t\right)}Z^{\prime }\lambda .$
(2) Compute GEBV by WssGBLUP (the first iteration is ssGBLUP).
(3) Calculate SNP effects by $\lambda D_{\left(t\right)}Z^{\prime }G_{\left(t\right)}^{*-1}GEBV.$
(4) Calculate SNP*_i_* weight by $CT^{\frac{\left|SNP\;effect_{i}\right|}{SD\left(SNP^{\prime }seffects\right)}\;-\;2}$ (nonlinear A weights method).
(5) Normalize $D_{\left(t+1\right)}=\frac{trace\left(D_{\left(t\right)}\right)}{trace\left(D_{\left(t+1\right)}\right)}D_{\left(t+1\right)}.$
(6) Calculate $G_{\left(t+1\right)}^{*}$ = $ZD_{\left(t+1\right)}Z^{\prime }\lambda .$
(7) *t* = *t* +1.
(8) Loop to 2 or exit if *t* >5.

where CT is a constant value that determines the departure from normal distribution. If CT equates to 1, it is the normal distribution; 1.050, 1.125 (default value), 1.500, and 2.000 were tested in this study. Previous studies have shown that citrate is strongly affected by a few genomic regions (Sanchez et al., 2021; Chen et al., 2024); therefore, the CT was set larger than 1. Five iterations were used in the study, which were used to optimize SNP weights and maximize accuracy gains (Cesarani et al., 2021).

Variance components and EBV (or GEBV) were estimated by using the BLUPF90+ (version 2.42) program (Misztal et al., 2014). Variance components were estimated through the average information restricted maximum likelihood estimation method (AI-REML). The EBV (or GEBV) was calculated through ABLUP (or ssGBLUP and WssGBLUP). The SNP effect and weight were calculated using POSTGSF90 software (version 1.73; Misztal et al., 2014).

A linear regression-based (**LR**) method developed by Legarra and Reverter (2018) was used to assess the prediction accuracy of the EBV (or GEBV) in young animals. The basic step of the LR method involves calculating the evaluation metrics by regressing the breeding value of the partial dataset according to the breeding value of the whole dataset. The pedigree and genome information of the whole and partial datasets were the same, but the phenotypic data were different. The phenotypes of the whole dataset were from 2012 to 2019, whereas the phenotypes of the partial dataset were from 2012 to 2016 (2017 to 2019 were set as missing values, n = 38,906). Both variance components and breeding values need to be estimated again in the partial dataset. The 181 youngest cows (born after 2015) with genotypic were selected for the validation population. Four following metrics were used to measure prediction validation results.

(1) Prediction accuracy $\left(\hat{acc}\right)$ of the validation population is expected to be 1 if the evaluation is perfect, as defined below:
  $$\hat{acc}=\;\sqrt{\frac{cov\left({\hat{u}}_{p},{\hat{u}}_{w}\right)}{\left(1-\bar{f}\right)\sigma_{u}^{2}}},$$
  where ${\hat{u}}_{p}$ and ${\hat{u}}_{w}$ are vectors of EBV (or GEBV) of the validation population in the partial and whole datasets, respectively; $\bar{f}$ is the average inbreeding coefficient of the validation population; and $\sigma_{u}^{2}$ is the additive genetic variance.
(2) Population bias (*µ_w,p_*) is expected to be 0 under an unbiased evaluation, as defined below:
  $$\mu_{w,p}=\bar{{\hat{u}}_{p}}-\bar{{\hat{u}}_{w}},$$
  where $\bar{{\hat{u}}_{p}}$ and $\bar{{\hat{u}}_{w}}$ are average (G)EBV of the validation population in the partial and whole datasets, respectively.
(3) Dispersion (*b_w_*_,_*_p_*) is expected to be 1 when there is no observed dispersion, as defined below:
  $$b_{w,p}=\;\frac{cov\left({\hat{u}}_{w},\;{\hat{u}}_{p}\right)}{var\left({\hat{u}}_{p}\right)},$$
  where all parameters are the same as described previously.
(4) Slope (*b_p_*_,_*_w_*) is expected to be 1 when the average reliability of the validation population is consistent between partial and whole datasets, as defined below:
  $$b_{p,w}=\frac{Reliability_{p}}{Reliability_{w}}=\;\frac{cov\left({\hat{u}}_{p},\;{\hat{u}}_{w}\right)}{var\left({\hat{u}}_{w}\right)},$$
  where all parameters are the same as described previously. The *b_p_*_,_*_w_* is used for assessing the relative stability in the average reliability of the validation population between estimates derived from partial and whole datasets. The data preparation and figure plot were performed using R (version 4.1.2, https://www.r-project.org/).

The average and standard deviation of citrate were 9.04 and 1.65 mmol/L, respectively. Table 1 shows the validated results from ABLUP and ssGBLUP for citrate. The $\hat{acc}$ and *b_p_*_,_*_w_* obtained from ssGBLUP increased by 65.19% and 85.28%, respectively, compared with those derived from ABLUP. The *µ_w,p_* and *b_w_*_,_*_p_* from ssGBLUP were similar to the results from ABLUP. Similar findings were also reported by Cesarani et al. (2021). This confirms that genomic information is very beneficial for the genetic evaluation of citrate.

**Table 1:** Validated predicted milk citrate by linear regression (LR) for best linear unbiased prediction with pedigree (ABLUP), single-step genomic BLUP (ssGBLUP, pedigree combined genomic; n = 181 youngest cows)

LR statistic	ABLUP	ssGBLUP
Prediction accuracy	0.42	0.70
Bias	0.04	−0.03
Dispersion	0.95	1.05
Slope	0.41	0.76

Figure 1 shows the validated results from WssGBLUP in the first 5 iterations. The $\hat{acc},$ *b_w_*_,_*_p_*, and *b_p_*_,_*_w_* of WssGBLUP (iterations 2–5) were better than those from ssGBLUP (first iterations); however, the *µ_w,p_* of WssGEBLUP was worse. The outcomes of WssGBLUP were evidently influenced by the CT value. The $\hat{acc}$ increased with increasing CT values, consistent with the finding that citrate was affected by a few genomic regions (Sanchez et al., 2021; Chen et al., 2024). The absolute *µ_w,p_* increased with increasing CT values. This may be due to the gradual increase in the mean GEBV of the absolute values across the validation population. The *b_w_*_,_*_p_* worsens as CT increases; however, the *b_w_*_,_*_p_* reached its best value (0.99 or 1.01) in the second (or third) iteration when CT was equal to 1.500 (or 1.250). The *b_p_*_,_*_w_* also increased with increasing CT values. The *b_p_*_,_*_w_* reached its best value (0.82) in the second iteration when CT was equal to 2.000. Based on the above results, the 4 metrics of WssGBLUP can be relatively good in the second iteration. Teissier et al. (2018) also reported that the maximum prediction accuracy was obtained at the second iteration. In the second iteration, 2 metrics $(\hat{acc}$ and *b_p_*_,_*_w_*) reached their best value when CT was equal to 2.000; however, another 2 metrics (*µ_w,p_* and *b_w_*_,_*_p_*) performed worst. Therefore, the CT of 1.500 was chosen as the best value in this study. Previous studies have also reported that WssGBLUP is more beneficial for GP compared with ssGBLUP (Teissier et al., 2018, 2019; Mehrban et al., 2021). However, if no significant genomic regions were associated with the studied trait, WssGBLUP may have similar results to ssGBLUP (Teissier et al., 2019; Cesarani et al., 2021).

**Figure 1:**
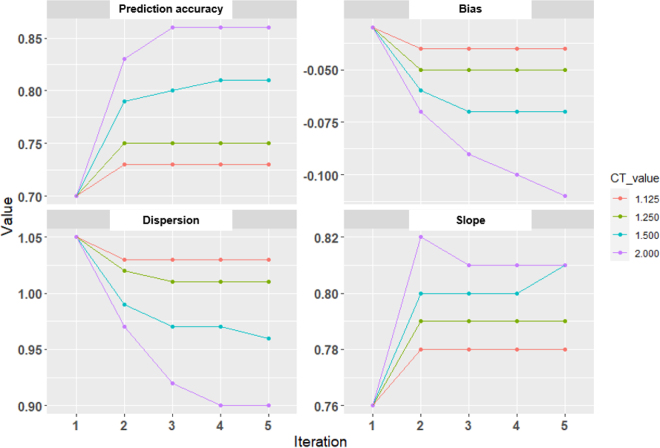
Validated predicted milk citrate by linear regression (LR) for weighted single-step genomic BLUP (WssGBLUP, pedigree combined genomic using SNP-weighted) in the 5 iterations with different CT values (n = 181 youngest cows). The weight of the SNP in the first iteration of all analyses was 1 (equated to ssGBLUP), so all results were the same. The CT values determine departure from normality for SNP effects (when CT equals 1, the SNP effect is normally distributed).

Some aspects of this research that can be improved in the future. The number of the validation population is small (n = 181). Therefore, more genotyped individuals are needed to be accumulated to further verify the results of this study. In contrast, we demonstrate that WssGBLUP is beneficial for the GP of small reference populations (local breeds). The WssGBLUP used in this study is limited to single-trait analysis and cannot perform multitrait analysis. Meuwissen et al. (2024) recently proposed an algorithm called GWABLUP: integrating GWAS results into GP. GWABLUP is capable of conducting multitrait analysis; however, it is currently being extended to single-step analysis. Assigning weight to SNPs can be a quick way to improve GP in the presence of genes with large effects. Weight estimation of SNP can be derived from a variety of information, currently mainly from SNP effects, and multi-omics information may contribute to this.

This study confirms that genomic information is beneficial for the genetic evaluation of citrate. The results of this study demonstrate that weighted SNP contributes to enhanced accuracy of GP for predicted milk citrate.
